# Houston hurricane Harvey health (Houston-3H) study: assessment of allergic symptoms and stress after hurricane Harvey flooding

**DOI:** 10.1186/s12940-021-00694-2

**Published:** 2021-01-19

**Authors:** Abiodun O. Oluyomi, Kristen Panthagani, Jesus Sotelo, Xiangjun Gu, Georgina Armstrong, Dan Na Luo, Kristi L. Hoffman, Diana Rohlman, Lane Tidwell, Winifred J. Hamilton, Elaine Symanski, Kimberly Anderson, Joseph F. Petrosino, Cheryl Lyn Walker, Melissa Bondy

**Affiliations:** 1 Department of Medicine, Section of Epidemiology and Population Sciences, Baylor College of Medicine, One Baylor Plaza, Jewish Building, Room 607D, (MS BCM307), Houston, TX USA; 2 Department of Family and Community Medicine, Environmental Health Service, Baylor College of Medicine, Houston, TX USA; 3 Genetics and Genomics, Baylor College of Medicine, Houston, TX USA; 4 Medical Scientist Training Program, Baylor College of Medicine, Houston, TX USA; 5 Center for Precision Environmental Health, Baylor College of Medicine, Houston, TX USA; 6 Department of Molecular Virology and Microbiology, Alkek Center for Metagenomics and Microbiome Research, Baylor College of Medicine, Houston, TX USA; 7 Environmental and Occupational Health, College of Public Health and Human Sciences, Oregon State University, Corvallis, OR USA; 8 Food Safety and Environmental Stewardship Program, Oregon State University, Corvallis, OR USA; 9 Molecular and Cellular Biology, Baylor College of Medicine, Houston, TX USA; 10 Department of Epidemiology and Population Health, Stanford Cancer Institute, Stanford University, Stanford, CA USA

**Keywords:** Disaster epidemiology, Environmental exposure assessment, Post-disaster rapid response research, Extreme weather events, Hurricanes, Flooding, Post-flooding environmental stressors, Post-flooding respiratory outcomes, Geographic information system

## Abstract

**Background:**

In August 2017, Hurricane Harvey caused unprecedented flooding across the greater Houston area. Given the potential for widespread flood-related exposures, including mold and sewage, and the emotional and mental toll caused by the flooding, we sought to evaluate the short- and long-term impact of flood-related exposures on the health of Houstonians. Our objectives were to assess the association of flood-related exposures with allergic symptoms and stress among Houston-area residents at two time points: within approximately 30 days (T1) and 12 months (T2) after Hurricane Harvey’s landfall.

**Methods:**

The Houston Hurricane Harvey Health (Houston-3H) Study enrolled a total of 347 unique participants from four sites across Harris County at two times: within approximately 1-month of Harvey (T1, *n* = 206) and approximately 12-months after Harvey (T2, *n* = 266), including 125 individuals who participated at both time points. Using a self-administered questionnaire, participants reported details on demographics, flood-related exposures, and health outcomes, including allergic symptoms and stress.

**Results:**

The majority of participants reported hurricane-related flooding in their homes at T1 (79.1%) and T2 (87.2%) and experienced at least one allergic symptom after the hurricane (79.4% at T1 and 68.4% at T2). In general, flood-exposed individuals were at increased risk of upper respiratory tract allergic symptoms, reported at both the T1 and T2 time points, with exposures to dirty water and mold associated with increased risk of multiple allergic symptoms. The mean stress score of study participants at T1 was 8.0 ± 2.1 and at T2, 5.1 ± 3.2, on a 0–10 scale. Participants who experienced specific flood-related exposures reported higher stress scores when compared with their counterparts, especially 1 year after Harvey. Also, a supplementary paired-samples analysis showed that reports of wheezing, shortness of breath, and skin rash did not change between T1 and T2, though other conditions were less commonly reported at T2.

**Conclusion:**

These initial Houston-3H findings demonstrate that flooding experiences that occurred as a consequence of Hurricane Harvey had lasting impacts on the health of Houstonians up to 1 year after the hurricane.

**Supplementary Information:**

The online version contains supplementary material available at 10.1186/s12940-021-00694-2.

## Background

On August 25, 2017, Hurricane Harvey, the second-costliest natural disaster in U.S. history [1–3] made landfall in Texas, and caused unprecedented catastrophic flooding across the greater Houston area, the nation’s fourth-largest city. Torrential rainfall over several days [4, 5] dropped an estimated 27 trillion tons of rain water on the Houston metropolitan area [6, 7], leading to the damage of roughly 136,000 homes [8] and causing 68 direct fatalities [9] and more than 75 total deaths [10, 11]. Houston’s Hobby Airport recorded 35.6 in. of rain from August 26–29, a record for wettest 4-day period for Houston [12].

Flooding overwhelmed numerous sewage treatment plants and triggered major releases of chemicals into the air from emergency shut-down and start-up procedures at area petrochemical facilities [13]. By September 2, 2017, the Environmental Protection Agency (EPA) determined that 13 of the 41 Superfund sites in the affected areas flooded and/or experienced possible damage due to the storm [14, 15], raising concerns among residents that homes and small businesses nearby or downstream may have been exposed to toxic chemicals. Environmental concerns remained after floodwaters receded as individuals participated in residential flood clean-up efforts, which took many months in most instances, potentially leading to long-term exposures to storm-related air pollution, contaminated water, debris, and mold. Additionally, this unprecedented flooding, which led to evacuations, injuries, financial stress, and lengthy displacement caused significant emotional trauma.

Although the literature focusing on the health consequences of disasters, including flooding, is growing, significant epidemiological gaps remain. In particular, allergic and psychological sequelae of natural disasters remain significant topics of interest warranting further inquiry. Recognizing the need to assess flood-related exposures and evaluate the impact of these exposures on area residents affected by Hurricane Harvey, we launched the Houston Hurricane Harvey Health Study (Houston-3H Study). This multi-component study was designed to evaluate flood-related exposures and to assess their impact on short- and long-term health outcomes. We report here the initial findings from the Houston-3H Study. In addition to analysis of the health data, other components of the Houston-3H Study that are being analyzed include 1) exposure to volatile and semivolatile organic chemicals, and 2) nasal, salivary and fecal samples. These analyses will, we hope, inform future disaster response activities to better protect human health. In addition, our experience and findings may lead to improved development and deployment of appropriate infrastructure for efficiently obtaining, storing and analyzing relevant biospecimens and data sets in similar disaster scenarios.

## Methods

### Study design and population

#### Study setting

The Houston-3H Study was a two-year multicomponent observational study that collected data at two time points: time 1 (T1) data were collected between September 21, 2017 and October 20, 2017. This was within 45 days of the end of Hurricane Harvey. Harvey officially ended on September 2, 2017. Time 2 (T2) data were collected at roughly 12–14 months after Harvey, between September 2018 and November 2018. By design, participation at T1 was not a prerequisite for T2 participation, though all T1 participants were invited to provide data at T2. Also, while certain questions were repeated at T1 and T2, others were collected at only one of the two time points. In the present study, to maximize statistical power for planned regression analyses, T1 data and T2 data were analyzed separately as cross-sectional data. Also, a secondary analysis assessed whether there were significant differences in the health conditions reported at T1 versus T2 exclusively among the participants that provided data at both times.

**Fig. 1:**
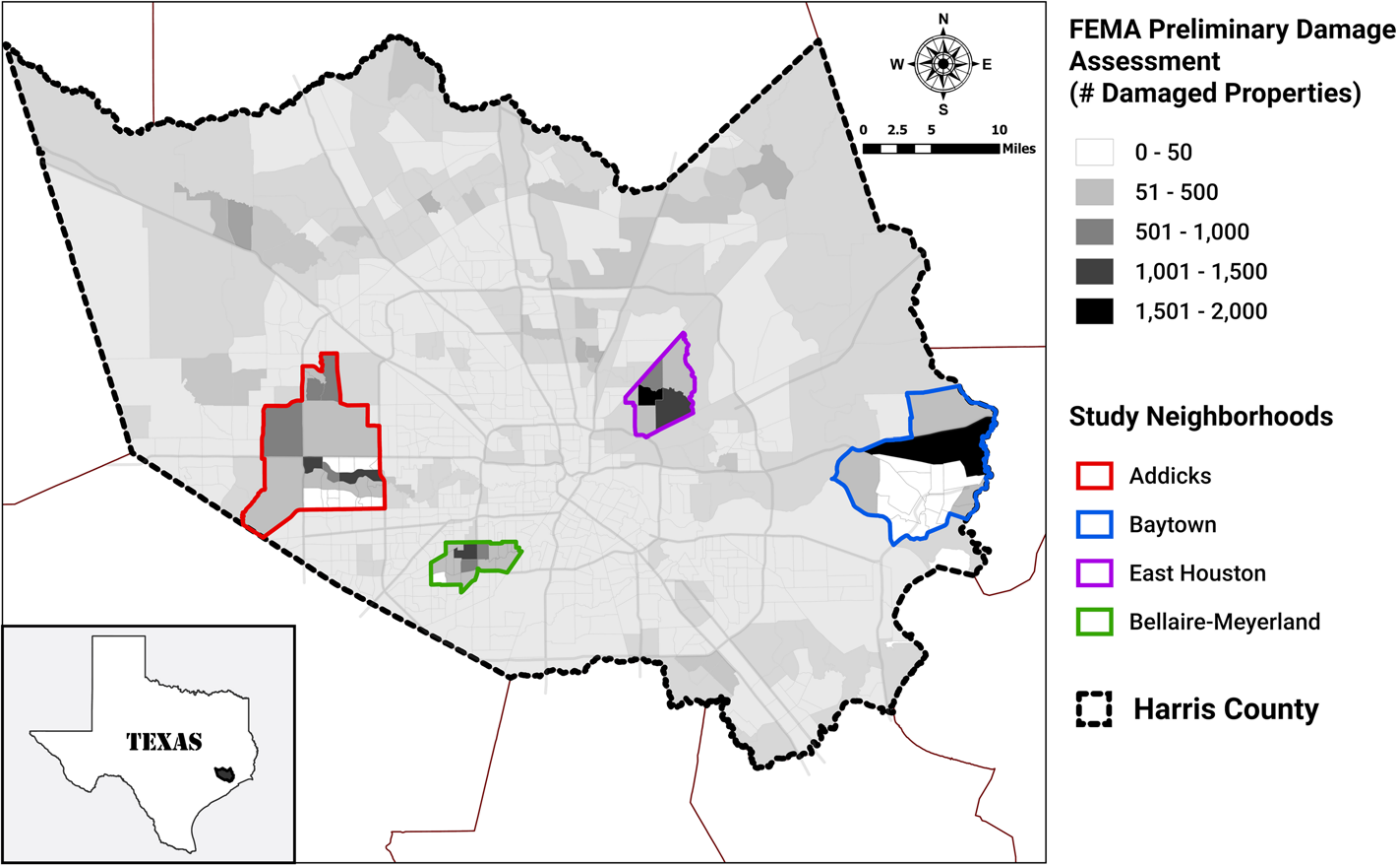
Original study neighborhoods based on Federal Emergency Management Agency preliminary damage assessment, Harris County, Texas

Our goal was to recruit participants from Harris County neighborhoods that were significantly impacted by the hurricane. Harris County, which contains the city of Houston, is the most populous county in Texas, with over 4.6 million people (Census, 2018). To assess impact, we obtained Federal Emergency Management Agency (FEMA) preliminary damage assessment (PDA) data on the number of hurricane-affected properties in each census tract in Harris County as of September 2, 2017. We used geographic information system (GIS) tools (ArcMap 10.5; Esri, Redlands, CA) to map FEMA’s PDA data and identify neighborhoods that had high counts of damaged properties. Subsequently, we worked with our community partners to identify a specific location within each neighborhood to use for recruitment and enrollment (Fig. 1). The selected neighborhoods and recruitment locations, in parentheses, were: Addicks (St. John Vianney Catholic Church), Baytown (Holy Family Catholic Church & JD Walker Community Center), and East Houston (East Houston Civic Club & Grace Cathedral Church). The Addicks and East Houston locations also served as disaster assistance centers after Harvey. In addition to these targeted neighborhoods, we also recruited participants at the Baylor College of Medicine (BCM) campus, the majority of whom were BCM employees who resided in and around the Bellaire-Meyerland neighborhood, also a FEMA high-impact neighborhood.

Each study neighborhood had different characteristics and Harvey-related exposures. The Addicks neighborhood, a relatively affluent Houston suburb, was primarily affected by reservoir flooding and by numerous sewage facilities that failed. Record inundation of the Addicks and Barker reservoirs caused flooding of approximately 9000 homes and businesses upstream [16], and thousands more downstream after purposeful release of water from the reservoirs to control upstream flooding and protect the integrity of the earthen dams [17]. Baytown is home of the nation’s largest refinery (the ExxonMobil Baytown Complex) and one of the largest chemical manufacturing complexes (the ChevronPhillips Cedar Bayou Plant). It is also downstream of the Highlands Acid Pits and San Jacinto River Waste Pits Superfund sites. The San Jacinto River Waste Pits experienced minor damage on the south berm crest, though post-storm assessments demonstrated no release of material to the environment [18]. East Houston is an older largely low-income inner-city neighborhood. East Houston residents were somewhat more likely to remain in their flood-damaged homes during clean-up and remediation because of limited options to relocate. Much of the Bellaire-Meyerland neighborhood, where most BCM enrollees lived, is located inside the 100-year floodplain [19] and is prone to flooding. Hurricane Harvey was the latest of three flooding events that inundated Meyerland in consecutive years, following the Memorial Day (2015) and Tax Day (2016) floods [20].

#### Participant recruitment and enrollment

Our ability to deploy within the critical 30-day window (T1) was enhanced by established or growing relationships with stakeholders in the selected recruitment locations. With a time-critical need to engage study participants during the T1 window, our research team pursued rapid IRB approvals from the different participating institutions. Leveraging and expanding an existing disaster IRB previously developed at Oregon State University (OSU), the BCM team worked with the BCM Institutional Review Board (IRB) office to obtain a rapid approval and the University of Texas Health Science Center (UTHealth) team received reciprocal approval. Within 7 days after BCM re-opened on August 30 (5 days after landfall), we had received all necessary IRB approvals to launch the study. All study materials were prepared in Spanish and English.

We worked cooperatively with key stakeholders at each recruitment location to publicize the study and invite people to participate. Our advertisement strategies included writing letters of introduction to community stakeholders, creating and sharing study flyers, and sending authorized study invitation emails to BCM listservs. In addition, BCM distributed press releases for study recruitment events that were advertised on local news, newspapers, and other print and web media. Eligibility criteria included individuals who were 5 years of age or older, fluently conversant in English or Spanish, and engaged in any form of Harvey-related cleanup. At each recruitment location, research team members explained the study to potential participants and obtained their written informed consent. At the BCM site, all BCM employees and students initially received an online screening questionnaire via email. Answers to the screening questionnaire were used to identify eligible individuals who were then invited to enroll. T1 recruitment, enrollment, and data collection activities began within 2 weeks of Hurricane Harvey and were completed within the following 30 days (the mean enrollment date was 1 month after Harvey). Time 2 (T2) fieldwork activities were two-pronged: first, all T1 participants who consented to a re-contact were invited to be re-assessed at 1 year, and second, a new set of participants were enrolled for the first time. All T2 activities occurred approximately 1 year (12 to 14 months) after Hurricane Harvey.

#### Data collection

After consenting, participants at both time points completed a health and exposure questionnaire and provided saliva and nasal swab samples on-site for oral and nasal microbiome analyses. Participants were also provided a take-home kit with (1) a silicone wristband for monitoring exposure to chemicals [21] (2) supplies for collecting a fecal sample (for gut microbiome analysis), and (3) a swab to obtain a surface home sample (for indoor microbiome analysis). The tests were discussed with the participants, and detailed instructions included in the kit. Arrangements were made for participants to return the wristband, swab, and fecal sample after 1 week. Participants received $10 cash at enrollment and $20 cash when they returned their wristband and other samples.

### Measures

The Houston-3H Study assessed four types of data: (1) self-reported questionnaire data; (2) microbiome data from nasal, saliva, and fecal samples and from swabs of the participant’s home; (3) chemical exposure data from the wristband passive samplers; and (4) area-level exposure and socioeconomic data from geospatial processing of secondary data. This paper reports the results of the analyses of the self-reported questionnaire data. Houston-3H Study’s questionnaire items were mostly drawn from the NIH Disaster Research Response (DR2) website (https://dr2.nlm.nih.gov/). In general, the questions asked about: (1) sociodemographic characteristics, (2) hurricane preparedness and response, (3) hurricane damage- and flood-related experiences, (4) cleanup activities and possible environmental exposures, (5) allergic and mental health outcomes, (6) hurricane financial impact, and (7) dietary and other health-related behaviors. 

#### Harvey-related exposures

At T1, Harvey exposure experience was obtained using the participants’ answers to seven questions: five single-item questions and two multi-item questions. The single-item questions asked participants whether: (1) their home flooded during Harvey; (2) they saw signs of mold, or smelled a moldy or musty odor during Harvey that was not there before Harvey; (3) they were rescued from their home during Harvey; (4) they suffered any injury within 30 days after Harvey; and (5) they were involved in flood clean-up efforts (*muck and gut*) during the 30 days after Harvey. The sixth question, which required a “yes/no” response to each of several items, was “In the first month after Hurricane Harvey, were you exposed to any of the following as far as you know?” The exposure items were: sewage, debris, dirty or contaminated floodwater, visible mold, exhaust fumes from generators, and diesel fuel or heating oil leaks. Participants could also indicate none of the listed exposures. The seventh question that also required a “yes/no” response to four items was: “Which of the following did you personally do at a home or homes damaged by Harvey, whether it was your home or someone else’s home?” The four referenced activities were: remove water, remove mud, tear out work, and major repair. The two questions about “mucking and gutting” (i.e., questions 5 and 7) were not included in the T2 questionnaire; the other questions were asked at both time points.

#### Allergic symptoms and stress

At T1, allergic symptoms were assessed using a series of yes/no responses to the question: “In the last 30 days, have you experienced any of these symptoms when you did NOT have a cold, the flu, or seasonal allergies?” At T2, “In the last 30 days” was replaced with “Since Harvey.” Symptoms asked about included: shortness of breath, wheezing, persistent cough, sinus problems/nose irritation, eye irritation, throat irritation, and skin rash/irritation. Notably, our questionnaires asked about any allergic symptoms since Harvey, which may have included symptoms that had since resolved. At T1, stress was measured with a “0 to 10” rating scale to the question: “How stressful overall would you say your experiences with Harvey and its aftermath have been?...where 0 means not at all stressful and 10 means the most stressful thing you can imagine.” At T2, the question was: “How stressed are you right now due to Harvey on a scale of 0-10 with 10 the most stressful?”

#### Covariates

Four covariates were included a priori in the regression analyses: (1) age calculated based on reported date of birth, (2) sex dichotomized into female or male, (3) race/ethnicity grouped into five categories: non-Hispanic black, non-Hispanic white, Hispanic, Asian, or Other (which included those that did not specify any race/ethnicity), and (4) education categorized as high school or less, some college, undergraduate, or advanced degree.

#### Area-level socioeconomic disadvantage

Given that residential neighborhoods have both direct and indirect effects on health [22–24], we performed exploratory analyses using the Area Deprivation Index (ADI) to reveal any roles that socioeconomic disadvantage might play in the relationships that we examined. The ADI is a composite measure of “neighborhood” socioeconomic disadvantage that is based on 17 U.S. Census measures from the following four categories: poverty, housing, employment, and education. We used the U.S. Census 2012–2016 American Community Survey (ACS) 5-year estimates data summarized to the census tract geographic level. We followed Singh’s formula [25, 26] to compute ADI scores for all the census tracts (*n* = 5265) in the state of Texas. We then used ArcGIS to geocode Houston-3H participants’ residential addresses during Hurricane Harvey, assigning to each participant the ADI score of the census tract in which they resided. For the 103 census tracts that contained all 347 participants, the median ADI score (106.23) was used to stratify all participants into low (ADI Score < 106.23) and high (ADI Score ≥ 106.23) area deprivation groups. Higher ADI scores represent greater socioeconomic disadvantage.

### Statistical analysis

All questionnaire data were coded, checked in batches by two assessors, and entered into the study database in Research Electronic Data Capture (REDCap) software. The cleaned dataset was imported into SAS (version 9.4, SAS Institute, Inc. Cary, NC) or R (version 3.6.1) for subsequent analyses. To reiterate, in the present study, the T1 data and T2 data were analyzed separately as cross-sectional data for all regression analyses. Thus, our emphasis is on the findings at each time period: immediately after the disaster and approximately 1 year after. For those who participated at both time points, their answers at T1 were used for T1 regression analysis, and answers at T2 were used for the T2 regression analysis. First, descriptive statistics were computed for relevant categorical variables (frequency distributions) and continuous variables (e.g., mean, standard deviation, median, and quartiles). At each time period, unconditional logistic regression was used to regress each allergic symptom (yes/no) on each Harvey exposure (yes/no) while adjusting for age, sex, race/ethnicity, and education level. Results of the regression models were used to calculate corresponding odds ratios, 95% confidence intervals, and *p*-values. Linear regression was used to regress stress level (scored from 0 to 10) on Harvey exposures (yes/no), adjusting for age, sex, race/ethnicity, and education level. All regression analyses were performed separately for T1 data and T2 data. Considering that multiple testing may increase type-1 error rate, we corrected for multiple comparisons using the false discovery rate adjusted (FDR-adjusted) p-values; i.e., q-values. For the exploratory analysis of neighborhood-level socioeconomic disadvantage, first, at each time period (T1 and T2, separately), we tested for differences in relevant variables between low and high ADI groups using chi-square, T, or Wilcoxon rank-sum tests as appropriate. We then performed the earlier described unconditional logistic and linear regression analyses on the ADI-stratified data set. However, to avoid issues with model convergence, we did not adjust for the race/ethnicity and education in the ADI-stratified analyses. To supplement the foregoing regression analyses, we assessed whether there were significant differences in the health conditions reported at T1 versus T2 exclusively among the participants that provided data at both times (the paired-samples; *N* = 125). We used the McNemar test to assess the null hypothesis that the proportion of the “yes” response to a particular health condition at T1 = proportion of the “yes” response at T2. Notably, symptoms reported at both time points may have included symptoms that had already resolved. For stress, we used the paired-samples t-test to determine whether the mean difference between paired observations is different from zero.

## Results

**Fig. 2:**
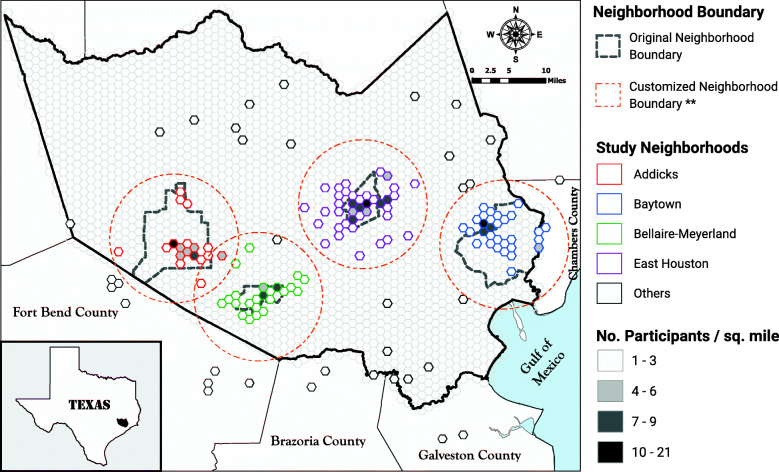
Location and concentration of Houston-3H Study participants in the Houston metro area, Harris County, Texas. ** We used the “Near” tool in ArcGIS Pro 2.1 to measure the Euclidean distance from each participant’s address to the nearest “original neighborhood” centroid (center). After visual inspection of address clustering around the centroid, a 7.5-mile radius was used as the cutoff for the “customized neighborhood boundary.” Most participants (*n* = 303) lived inside the customized neighborhood boundaries. Those who lived outside the customized neighborhoods were assembled into an “Others” group (*n* = 44)

The distribution of study participants across the four study neighborhoods and other parts of the Houston metro area is shown in Figs. 1 and 2. A total of 347 unique participants were enrolled in our study, with 206 participants within approximately 1 month of Hurricane Harvey (T1 data), followed by 266 participants at approximately 12 months post-Harvey (T2 data). Of the 266 T2 participants, 125 were repeat participants—those that provided data at T1 and T2 (Fig. S1). By enrolling at multiple study locations across Harris County, this study captured participants with differing racial, ethnic, educational, and socioeconomic backgrounds, reflecting some of the diversity characteristic of the Houston region. The distributions of sex, age, race/ethnicity, and educational attainment at each time point are provided in Table 1 (T1 data) and Table 2 (T2 data).

**Table 1:** Demographic, environmental & health characteristics of all participants at time point 1 (T1), (*N* = 206)

Characteristics	N	(%)
**SOCIODEMOGRAPHIC**		
**Sex**		
Female	148	(71.8)
Male	58	(28.2)
**Age (yrs.)**		
Younger than 18	17	(8.2)
18 to 34	28	(13.6)
35 to 44	28	(13.6)
45 to 64	89	(43.2)
65 or older	44	(21.4)
*Missing*	*0*	
**Race/Ethnicity**		
Asian	16	(7.8)
Black (NH)^a^	83	(40.5)
Hispanic	40	(19.5)
Other Not Specified	6	(2.9)
White (NH)^a^	60	(29.3)
*Missing*	*1*	
**Education**		
High School or less	54	(27.1)
Some College	32	(16.1)
Undergraduate	62	(31.2)
Advanced Degree	51	(25.6)
*Missing*	7	
**ENVIRONMENTAL EXPOSURES**		
**Home Flooded**		
No	43	(20.9)
Yes	163	(79.1)
*Missing*	*0*	
**Signs of Household Mold**		
No	30	(18.0)
Yes	137	(82.0)
*Missing*	*39*	
**Individual Exposures (Yes)**^**b**^		
Sewage	100	(52.9)
Debris	129	(68.3)
Contaminated Waters	143	(75.7)
Mold	115	(60.8)
Exhaust Fumes from Generators	34	(18.0)
Leaks/Spills; Diesel Fuel/Heating	15	(7.9)
None of the Above	18	(9.5)
**Rescued during Harvey**		
No	135	(71.0)
Yes	55	(29.0)
*Missing*	*16*	
**Involved in Clean-Up**^**c**^		
No	58	(28.1)
Yes	132	(64.1)
*Missing*	*16*	*(7.8)*
**Activity in Flooded Homes (Yes)**^**c,d**^		
Remove Water	116	(56.3)
Remove Mud	118	(57.3)
Tear Out Work	112	(54.4)
Major Repair	26	(12.6)
None of the Above	23	(11.2)
**HEALTH OUTCOMES**		
**Stress Level Post-Harvey**		
Mean (SD)^e^	8.0	2.10
Median [IQR]^f^	8.0	[7, 10]
**Injuries due to Harvey**		
No	125	(68.3)
Yes	58	(31.7)
*Missing*	*23*	
**Allergic Symptoms**		
No	37	(20.6)
Yes	143	(79.4)
*Missing*	*26*	
**Symptoms since Harvey (Yes)**^**g**^		
Shortness of Breath	46	(25.8)
Wheezing	37	(20.2)
Persistent Cough	63	(34.8)
Sinus Problems	99	(55.9)
Eye Irritation	88	(49.2)
Throat Irritation	84	(48.0)
Skin Rash	46	(26.1)

Note: “Missing” shown for reference. Missing data not included in statistical analyses

^a^*NH* Not Hispanic/Latino

^b^Each individual exposure is not mutually exclusive. Participants were asked to check all that applied

^c^Items not included in T2 questionnaire

^d^Each individual activity is not mutually exclusive. Participants were asked to check ll that applied

^e^Standard deviation from mean stress level

^f^Interquartile range from mean stress level

^g^Each individual symptom is not mutually exclusive. Participants were asked to check all that applied

**Table 2:** Demographic, environmental & health characteristics of all participants at time point 2 (T2), (*N* = 266)

Characteristics	N	(%)
**SOCIODEMOGRAPHIC**		
**Sex**		
Female	174	(65.4)
Male	92	(34.6)
**Age (yrs.)**		
Younger than 18	25	(9.6)
18 to 34	26	(10.0)
35 to 44	41	(15.7)
45 to 64	98	(37.5)
65 or older	71	(27.2)
*Missing*	*5*	
**Race/Ethnicity**		
Asian	18	(6.8)
Black (NH)^a^	87	(32.7)
Hispanic	50	(18.8)
Other Not Specified	5	(1.9)
White (NH)^a^	106	(39.8)
*Missing*	*0*	
**Education**		
High School or less	75	(28.7)
Some College	31	(11.9)
Undergraduate	74	(28.4)
Advanced Degree	81	(31.0)
*Missing*	*5*	
**ENVIRONMENTAL EXPOSURES**		
**Home Flooded**		
No	34	(12.8)
Yes	231	(87.2)
*Missing*	*1*	
**Signs of Household Mold**		
No	71	(33.3)
Yes	142	(66.7)
*Missing*	*53*	
**Individual Exposures (Yes)**^**b**^		
Sewage	148	(58.3)
Debris	188	(74.0)
Contaminated Waters	195	(77.4)
Mold	177	(70.5)
Exhaust Fumes from Generators	62	(24.7)
Leaks/Spills; Diesel Fuel/Heating	28	(11.2)
None of the Above	26	(10.2)
**Rescued during Harvey**		
No	138	(53.7)
Yes	119	(46.3)
*Missing*	*9*	
**HEALTH OUTCOMES**		
**Stress Level Post-Harvey**		
Mean (SD)^c^	5.1	3.20
Median [IQR]^d^	5.0	[2, 8]
**Injuries due to Harvey**		
No	178	(73.9)
Yes	63	(26.1)
*Missing*	*25*	
**Allergic Symptoms**		
No	80	(31.6)
Yes	173	(68.4)
*Missing*	*13*	
**Symptoms since Harvey (Yes)**^**e**^		
Shortness of Breath	63	(25.2)
Wheezing	61	(24.5)
Persistent Cough	68	(27.2)
Sinus Problems	124	(49.6)
Eye Irritation	99	(39.6)
Throat Irritation	85	(34.4)
Skin Rash	53	(21.5)

Note: “Missing” shown for reference. Missing data not included in statistical analyses

^a^NH – Not Hispanic/Latino

^b^Each individual exposure is not mutually exclusive. Participants were asked to check all that applied

^c^Standard deviation from mean stress level

^d^Interquartile range from mean stress level

^e^Each individual symptom is not mutually exclusive. Participants were asked to check all that applied

Assessment of Harvey-related environmental exposures at T1 (Table 1) revealed that the majority (79.1%) of study participants experienced Harvey-related flooding in their homes and saw signs of mold inside their homes after the hurricane (82.0%). The most common environmental exposures reported were exposure to: contaminated waters (75.7%), debris (68.3%), visible mold (60.8%), and sewage (52.9%). Less common exposures were fumes from generators (18.0%) and leaks/spills from diesel fuel or heating (7.9%). At T2 (Table 2), 87.2% of participants experienced Harvey-related flooding and 66.7% saw signs of mold inside their homes after the hurricane. The most common environmental exposures were exposure to: contaminated waters (77.4%), debris (74.0%), visible mold (70.5%), and sewage (58.3%). See Tables 1 and 2 for more details.

**Fig. 3:**
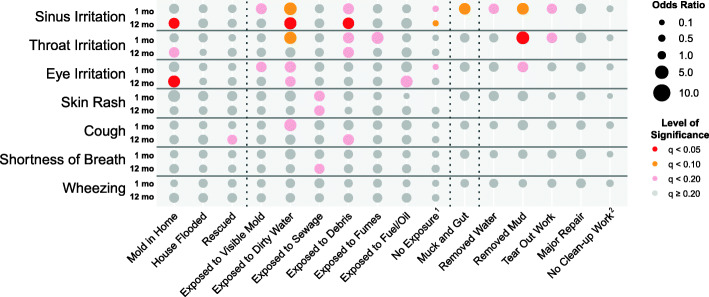
Logistic regression (Odds Ratio) of allergic symptoms on flooding exposures; time point 1 (*N* = 206) and time point 2 (*N* = 266). *Note (i):* Odds ratios are indicated by the size of the circle, and colors indicate level of significance after false discovery rate (FDR) correction. ^1^ No exposure refers to preceding six exposures; subjects indicating “No Exposure” were not exposed to visible mold, dirty water, sewage, debris, fumes, or fuel/oil. ^2^ No Clean-up Work refers to four previous work experiences; subjects indicating “No Clean-up Work” did not remove water, remove mud, perform tear out work, or perform a major repair

Using T1 data, symptoms experienced within 30–45 days of Harvey, 79.4% of participants reported at least one allergic symptom (Table 1). The most common allergic symptoms reported were upper respiratory tract symptoms including sinus irritation (55.9%), eye irritation (49.2%), and throat irritation (48.0%). Logistic regression of allergic symptoms and flooding exposures revealed that only a subset of flooding exposures assessed were associated with increased risk of allergic symptoms (Fig. 3, Table S1.1). In general, flooding exposures were associated with higher risk of upper respiratory tract symptoms, with fewer significant associations detected with lower respiratory tract symptoms and skin rash. Exposure to visible mold (anywhere) was associated with increased risk of sinus irritation (OR 2.69, 95% CI: 1.33—5.56) and eye irritation (OR 2.38, 95% CI: 1.19—4.89) only in the T1 cohort. Dirty water was associated with increased risk for the greatest number of allergic symptoms overall, including the following at T1: sinus irritation (OR: 4.11, 95% CI: 1.70—10.47), throat irritation (OR 3.79, 95% CI: 1.56—9.80), eye irritation (OR: 2.99, 95% CI: 1.25—7.47), and cough (OR: 3.75, 95% CI: 1.46—10.59).

Findings from T2 data also showed a high prevalence of allergic symptoms (symptoms experienced since Harvey), with the 68.4% of participants reporting at least one allergic symptom (Table 2). Upper respiratory tract symptoms remained the most common allergic symptoms reported, including sinus irritation (49.6%), eye irritation (39.6%), and throat irritation (34.4%). Again, positive associations were detected between flooding exposures and risk of upper respiratory tract symptoms (Fig. 3, Table S1.1). For example, exposure to mold in the home was associated with sinus irritation (OR: 2.67, 95%, CI: 1.41—5.15), throat irritation (OR: 2.33, 95%, CI: 1.17—4.84), and eye irritation (OR: 3.49, 95%, CI: 1.76—7.20). At T2, dirty water was associated with increased risk of sinus irritation (OR: 3.71, 95%, CI: 1.76—8.21), and eye irritation (OR: 2.37, 95% CI: 1.11—5.33).

**Fig. 4:**
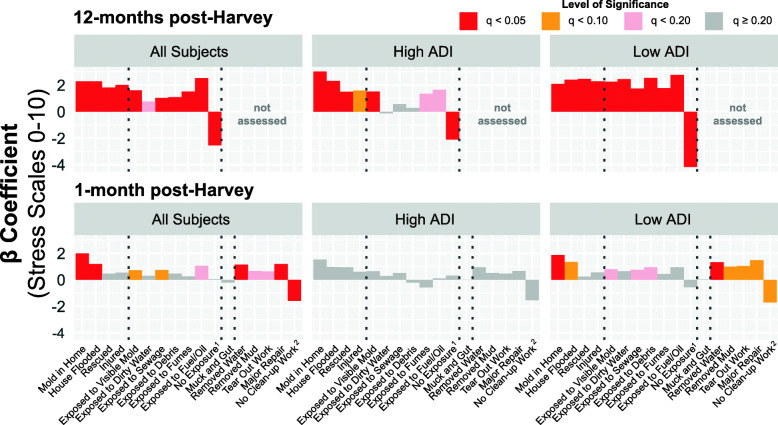
Linear regression of stress level (0 to 10) on flooding exposures; time point 1 (*N* = 206) and time point 2 (*N* = 266). *Note (i):* Area Deprivation Index (ADI) is a composite measure of “neighborhood” socioeconomic disadvantage. Higher score represents increasing socioeconomic disadvantage. *Note (ii):* Linear regression results for all study participants (left), high ADI participants (middle), and low ADI participants (right). *Note (iii):* Colors indicate level of significance after false discovery rate (FDR) correction. ^1^ No exposure refers to preceding six exposures; subjects indicating “No Exposure” were not exposed to visible mold, dirty water, sewage, debris, fumes, or fuel/oil. ^2^ No Clean-up Work refers to four previous work experiences; subjects indicating “No Clean-up Work” did not remove water, remove mud, perform tear out work, or perform a major repair

The mean reported stress level at T1 (rated on a 0—10 scale) was 8.0 (± 2.1), (Table 1). Linear regression of stress score and flooding exposures revealed that only a few exposures were associated with increased stress at T1 (Fig. 4, Table S1.2). At T1, participants that had significantly higher stress levels than their counterparts included: those that reported mold inside their homes (β = 1.99; *q* < 0.001), those whose homes flooded (β = 1.20; q = 0.014), those exposed to visible mold (β = 0.72; q = 0.076), those exposed to sewage (β = 0.74; q = 0.057), those involved in removing water from any flooded homes (β = 1.14; q = 0.002), and those involved in major Harvey-related repairs (β = 1.20; q = 0.018). Notably, participants who reported no clean-up activities had significantly lower stress level (β = − 1.59; q = 0.012).

For Harvey-related stress at roughly 12 months post-disaster, using T2 data, the mean reported stress level was 5.1 (± 3.2) on a 0–10 scale (Table 2). At T2 (Fig. 4, Table S1.2), participants who reported any exposure, except for exposure to dirty water, had significantly higher stress levels than their counterparts (at *q* < 0.05). Highest stress differentials occurred among those who reported: any exposure to visible mold (β = 1.61; *q* < 0.001), flooded homes (β = 2.29; *q* < 0.001), mold inside the house since Harvey (β = 2.30; *q* < 0.001), and exposure to fuel/oil (β = 2.52; *q* < 0.001). Participants who did not experience any of the listed exposures had significantly lower stress levels (β = − 2.54; *q* < 0.001) when compared to those who reported exposures.

Findings from our exploratory analysis that explored neighborhood disadvantage effects are reported for each time point. Comparing ADI groups at both time points (Tables S2.1 and S2.2), the majority of participants in the high ADI group were either non-Hispanic black or Hispanic (91.4% at T1 and 90.2% at T2), had lower educational attainment (67.01% at T1 and 73.1% at T2 less than a bachelor’s degree), and were younger (30.8% at T1 and 34.2% at T2 younger than 35 years of age) when compared with the low ADI group. In general, the proportion of participants whose homes flooded was higher among the low ADI group compared with the high ADI group, especially at T2 (90.9% in low ADI, 82.8% in high ADI). At both T1 and T2, environmental exposures were generally higher in the low ADI group compared with the high ADI group. For example, at T1, exposures among low vs. high ADI groups were: 75.8% vs. 44.4% (mold); 78.8% vs. 56.7% (debris); and 87.9% vs. 62.2% (contaminated waters). Skin rash was the only allergic symptom that differed between low ADI group (34.4%) and high ADI group (16.3%). We observed lower mean stress score among the low ADI group (7.7 ± 2.0) relative to the high ADI group (8.3 ± 2.22) at T1; this difference approached significance (*p* = 0.061). At T2, we observed no difference in the mean stress scores (low was 4.92 ± 3.28; high was 5.32 ± 3.20; *p* = 0.325). More details are provided in Table S2.1 and Table S2.2).

Overall, the ADI status had limited influence on the relationships between flooding exposures and the risk of allergic symptoms (Fig. S2, Table S3.1). At T1, those in the low ADI group had increased risk of sinus irritation after mucking and gutting (OR: 5.95, CI: 2.21—17.67) as compared with those in the high ADI group (OR: 1.56, CI: 0.56—4.38). At T2, those in the high ADI group who reported mold in the home had increased risk of throat irritation, eye irritation, wheezing, and cough (OR range: 4.83—7.44, *q* < 0.20) whereas those in the low ADI group did not (OR range: 1.0—2.74, *q* > 0.20). Although our analysis found little difference in overall stress between the ADI groups (Table S2), the low ADI group had significantly more exposures that were associated with increased stress than did the high ADI group at both T1 and T2 (Fig. 4, Table S3.2). Notably, in the high ADI group, no exposures were significantly associated with increased stress at T1. In the low ADI group, situations where T2 stress levels increased by at least 50% when compared with the T2 pooled data were: “exposed to sewage” (β = 1.75 vs. 1.04 for pooled) and “exposed to debris” (β = 2.55 vs. 1.10 for pooled). Estimated impact on stress decreased by more than 50% for “no Harvey exposures” in the low ADI group (β = − 4.16) versus pooled data (β = − 2.54).

For the paired-samples comparisons, out of the seven dichotomous (yes/no) health outcomes assessed, the McNemar’s test determined that the difference in the proportion of “yes” response at T1 and T2 was statistically significant for each of the following health outcomes, with less “yes” at T2: cough (*p* = .011), eye irritation (*p* = 0.009), sinus irritation (*p* = 0.026), and throat irritation (*p* = 0.035). The McNemar’s test retained the null hypothesis for shortness of breath, wheezing, and skin rash, that is, there was no significant difference in the proportions of the “yes” response to these conditions between T1 and T2. For stress, there was a statistically significant mean decrease of − 3.321 ± 2.99 (*p* < o.oo1). Detailed results of the paired samples analyses are shown in the Paired Samples Supplementary File.

## Discussion

Facilitated by established relationships across multiple academic institutions, rapid inter-institutional collaboration, and participation of key stakeholders from the targeted communities, we were able to launch this study of Hurricane Harvey-related exposures and health outcomes exceptionally quickly, allowing us to gather data in the immediate aftermath of Harvey (T1) and again roughly 12 months later (T2). In this initial analysis, we chose to focus on allergic symptoms and stress, by flooding exposure and area deprivation, using a cross-sectional design that ignores—for now—the issue of pairing in the subset of 125 individuals that provided data at both time points. We found that that specific flood-related exposures were associated with increased risk of allergic symptoms and increased stress levels immediately after and up to 1 year following Hurricane Harvey. These results add to the body of knowledge linking flooding experience with health outcomes. A previous review article, for example, by Alderman and associates found that respiratory symptoms not specific to any disease are common following flooding disasters [27]. They also noted that major flooding events can have long-term impacts on the health and well-being of affected individuals.

Earlier research has linked flooding to elevated exposures to such things as mold [28], hazardous chemicals [29, 30], and sewage [31], but the effects of such exposures on health has often been unclear [28, 29]. Barbeau and colleagues, for example, studied Hurricanes Katrina and Rita in the U.S. and found that mold growth was higher in homes with more severe flooding damage but that the mold was not associated with an increase in adverse health outcomes [28]. However, a cross-sectional study of the 2011 floods in Brisbane, Australia, found that residents affected by flooding were about twice as likely to report poor respiratory health [32], and a study of a flooding incident in Japan found that flooding significantly increased the incidence of respiratory, ocular, dermal, and nasal symptoms after the event [33]. In a study of households 4 months after a flood in El Paso, Texas, those who experienced flooding damage reported suffering from allergies, headaches, and irritation to the throat, nose, eyes and skin [34].

Our study is generally consistent with these findings but explored in greater detail the relationship between allergic symptoms and specific exposures. In our study, exposure to dirty water appeared to be one of the strongest risk factors for allergic health-related outcomes. Exposure to dirty water was associated with an increased risk of allergic symptoms including sinus, throat, and eye irritation, and cough. Although dirty water was associated with increased risk of allergic symptoms at both time points, the exposure-symptom association for some symptoms was stronger either immediately after the flooding (T1) or considerably later (T2). For example, exposure to visible mold (inside or outside the home) was associated with an increased risk of sinus and eye irritation within 1 month of Harvey, whereas signs of mold growth in the home were more strongly associated with these symptoms within 12-months of the hurricane. This suggest that mold inside the home, a potentially chronic exposure, can result in long-term allergic problems. Also, in the exploratory analysis of neighborhood disadvantage, at the later time point, findings suggest that signs of mold inside the home were associated with allergic symptoms (throat irritation, eye irritation, cough, wheezing) in the high ADI group but not the low ADI group. This may indicate that mold growth in the home may be particularly challenging for individuals in the high ADI neighborhoods.

In addition to physical ailments, psychological effects remain a significant public health concern for disaster research [35]. Others have reported a variety of psychological impacts following flooding, including posttraumatic stress disorder (PTSD), depression, and anxiety [36]. A study of severely flooded locales in Japan between 2004 and 2010 reported significantly higher incidence of post-traumatic stress disorder 6 months after the flooding among individuals with flooded homes [33]. Another study of children in Galveston, Texas, found that Hurricane Ike was associated with a significant increase in post-traumatic stress and sedentary activity among the children [37]. Our results are consistent with these previous studies demonstrating that flooding can have a long-term impact on mental health.

In our analysis of stress-exposure relationships, we discovered that while mean stress was lower at T2, specific exposures were associated with longer-term increases in stress. This suggests that short-term elevated stress from the disaster may have been somewhat universal, whereas longer-term stress may be associated with specific exposures or experiences during disasters. For example, at T1, we found no significant difference in stress levels between participants who marked “No Exposure” to visible mold, dirty water, sewage, debris, fumes, and fuel/oil compared with those who were exposed to at least one of these. At T2, however, participants who marked “No Exposure” reported significantly lower stress levels than those who experienced at least one of these exposures. In addition, these exposure-stress associations remained even after stratifying by neighborhood deprivation, especially at T2. However, there were differences in the observed effect estimates between the two ADI groups, with more exposures significantly associated with higher stress levels in the low ADI group compared to the high ADI group. This is an interesting finding that has not been previously reported, to the best of our knowledge, and warrants additional study. One hypothesis is that, perhaps, high ADI individuals may have developed resilience that low ADI individuals do not have. Other situations might be at play, e.g., high ADI individuals might have rented (vs. owned) their homes, and therefore did not lose a large portion of their financial net worth since most homeowner insurance policies do not cover flood damage.

The Houston-3H Study has several important strengths. First, we were able to collect extensive survey and biomarker data extremely quickly after Harvey made landfall, within 30 days after the hurricane in most instances. Few research teams have been able to mobilize this quickly. Second, we were able to collect T2 data 12 months later on 60% (*N* = 125) of the T1 participants, all of whom were recruited at T1 during the height of the disaster response and many of whom were displaced or in other ways underwent major Harvey-related changes in the subsequent 12 months. Whereas, few disaster studies have been able to collect data at these key time points, longitudinally, many environmentally-related health impacts can only be determined through longitudinal studies, [38]. Among our paired samples, findings from the comparisons of T1 versus T2 health conditions suggest that over the one-year follow-up period, conditions associated with breathing difficulty may have persisted longer than conditions associated with irritations and cough. The overall stress level reduced among this group, but skin irritation remained unchanged, perhaps an indication of longer-term effects from dermal exposures during Harvey. However, as subjects may have reported allergic symptoms that were ongoing or had already resolved, the temporality of these relationships cannot be conclusively determined. The current paired-samples findings supplement the rest of our work in this paper. Further longitudinal analysis beyond the scope of the current work that utilizes the Houston-3H paired-samples data more elaborately are forthcoming. Third, few studies have taken environmental samples from flooded homes [39] or examined short- and long-term exposures due to flooding and likely health effects [40]. Although a personal passive sampling post-disaster report has been published [41], the Houston-3H study is the first large-scale deployment of personal passive samplers measuring exposure to over 1500 volatile and semi-volatile organic chemicals. Fourth, we are unaware of any studies that have collected key microbiome biomarkers at multiple time points post flooding for subsequent genomic analysis. This is a strength of the overall study, although these data were not included in the current analysis. Results from microbiome samples and personal passive samplers will be reported in upcoming publications. Last, our study collected data from four unusually socioeconomically diverse communities in the Houston region, each of which experienced severe flooding. These characteristics of the Houston-3H Study and the large amount of data collected will, we hope, lead to a number of useful contributions, including the present report, to the field of disaster research and to “the overarching goals of preparedness and response: preventing injury, illness, disability, and death” [42].

The limitations of our study include the following. First, due to the rapid response nature of our study we restricted our sampling frame to specific locations and, thus, our results may not be generalizable to all communities in Houston. However, the inclusion of multiple neighborhoods with differing racial, ethnic, and socioeconomic populations mitigates this limitation to a degree, as it enables our study to reflect more of the diversity of Houston than would a study of a single locale. Second, the urgency for rapid deployment after Harvey limited our ability to fully develop and assess some of our collection methods. For example, some undoubtedly useful questions were not included in the T1 questionnaire, including household income and whether or not the participant had flood insurance. Third, as we were unable to follow-up with every T1 participant at 12-months post-Harvey, subtle demographic differences exist between the T1 and T2 cohorts, including a higher proportion of participants with advanced degrees at T2. This may impact resources available for flood remediation efforts and healthcare access in these participants, which could in turn alter health outcomes. Fourth, a higher percentage of participants in the high ADI group did not answer questions detailing exposures and allergic symptoms. This may have affected comparisons made between the two ADI groups. Finally, some participants lived in flood-prone areas that had been impacted by previous flooding events prior to Harvey; these previous exposures may have influenced these participants’ sensitivity to flood-related allergens such as mold as well as their stress response to Harvey flooding.

Although nearly all disasters carry a substantial public health risk and require both immediate and long-term assessment of their health effects on the population, significant challenges remain in the disaster epidemiology arena. For example, Miller and colleagues [38] described four key disaster research challenges, including: (1) lack of coordination of multiple stakeholders in identifying and prioritizing data gaps and post-disaster research questions; (2) research process challenges like IRB approval and funding; (3) research infrastructure supporting, for example, logistics and data collection; and (4) effective engagement with stakeholders in implementing research and reporting results. The Houston-3H study encountered several of these challenges to varying degrees. Based on our experience, we recommend that institutions in disaster-prone areas establish an ongoing multi-institutional disaster study protocol, which would enable researchers to immediately launch studies after disasters strike. This would include previously designed and vetted demographic, exposure, and symptom questionnaire. We also recommend that a draft informed consent document be prepared and tentatively approved by the IRBs in the most common languages in the study area, and that the consent include permission to re-contact participants to facilitate longitudinal follow-up, to obtain specific biomarkers, and to collect environmental samples. These recommendations are in line with a recent observation that the National Institute of Environmental Health Sciences (NIEHS) P30 Core Centers across the U.S. are in a position to leverage their scientific assets and community partnerships for disaster-related health outcomes research [43].

## Conclusions

The preliminary results of our Houston-3H study demonstrate that not all flooding experiences are identical, and that many factors, both disaster-related (such as type of flood-related exposure), and demographic (such as neighborhood ADI), may significantly impact post-disaster health outcomes. Our results support the findings of other disaster epidemiology studies which showed respiratory and psychological sequelae post flooding disasters. Importantly, the data presented here provide the most detailed assessment to date on the relationship between specific hurricane- or flooding-related exposures, allergic symptoms and stress levels. Future analyses of Houston-3H study data will provide additional insights into how other exposures, such as microbial and chemical exposures, interact with flood-related exposures, and possibly contribute to post-Hurricane Harvey health outcomes.

## Supplementary Information

**Additional file 1: Supplementary Table S1.1.** Environmental exposures and risk of allergic symptoms after Hurricane Harvey. Results: Unconditional logistic regression was used to regress allergic symptoms (yes/no) on Harvey exposures (yes/no). T1 and T2 data are presented side-by-side. **Supplementary Table S1.2.** Environmental exposures and stress burden after Hurricane Harvey. Results: Linear regression was used to regress stress level measure (0 to 10) on Harvey exposures (yes/no). T1 and T2 data are presented side-by-side.

**Additional file 2: Table S2.1.** Characteristics of study participants at time point 1 (T1) stratified by Area Deprivation Index (ADI). Results: Summary statistics displaying variable distribution, mean, standard deviation, median, and 1st and 3rd quartiles. We tested for differences between low and high ADI groups using chi-square, T, or Wilcoxon rank-sum tests as appropriate. Analysis performed on Area Deprivation Index-stratified (ADI-stratified) T1 data (Low ADI, *N* = 102); High ADI, *N* = 104). **Table S2.2.** Characteristics of study participants at time point 2 (T2) stratified by Area Deprivation Index (ADI). Results: Summary statistics displaying variable distribution, mean, standard deviation, median, and 1st and 3rd quartiles. We tested for differences between low and high ADI groups using chi-square, T, or Wilcoxon rank-sum tests as appropriate. Analysis performed on Area Deprivation Index-stratified (ADI-stratified) T2 data (Low ADI, *N* = 143); High ADI, *N* = 123).

**Additional file 3: Supplementary Table S3.1.** Environmental exposures and risk of allergic symptoms after Hurricane Harvey, stratified by ADI. Results: Unconditional logistic regression was used to regress allergic symptoms (yes/no) on Harvey exposures (yes/no). Findings presented separately for participants in Low ADI and High ADI neighborhoods. T1 and T2 data are presented side-by-side. **Supplementary Table S3.2.** Environmental exposures and stress burden after Hurricane Harvey, stratified by ADI. Results: Linear regression was used to regress stress level measure (0 to 10) on Harvey exposures (yes/no). Findings presented separately for participants in Low ADI and High ADI neighborhoods. T1 and T2 data are presented side-by-side.

**Additional file 4: Figure S1.** Houston Hurricane Harvey Health (Houston3H) Study flow of participants’ enrollment and data collection. A chart that shows how participants were assembled into the Houston-3H Study. **Figure S2.** ADI-stratified logistic regression (Odds Ratio) of allergic symptoms on flooding exposures at time point 1 (T1, *N* = 206) and time point 2 (T2, *N* = 266). A graphical representation of the results of the unconditional logistic regression used to regress allergic symptoms (yes/no) on Harvey exposures (yes/no). Figure depicts size of odds ratios and level of significance seperately for Low ADI vs. High ADI neighborhoods.

**Additional file 5. **Comparisons of the health outcomes reported by the participants that provided data at both T1 and T2 (*N* = 125). Several graphs and analysis output tables show the results from the analysis that comapred heath conditions at T1 versus T2 exclusively among the participants with repeated measures (the paired-samples; *N* = 125).

## Data Availability

Study materials including the questionnaires and the datasets used and/or analyzed during the current study are available from the corresponding author on reasonable request.
