# Supplementary material for: Houston hurricane Harvey health (Houston-3H) study: assessment of allergic symptoms and stress after hurricane Harvey flooding
Source: Environ Health. 2021 Jan 19;20:9. doi: 10.1186/s12940-021-00694-2 (PMC7816385; doi:10.1186/s12940-021-00694-2)
Supplement: Supplementary file 4 — Additional file 4: Figure S1. Houston Hurricane Harvey Health (Houston3H) Study flow of participants’ enrollment and data collection. A chart that shows how participants were assembled into the Houston-3H Study. Figure S2. ADI-stratified logistic regression (Odds Ratio) of allergic symptoms on flooding exposures at time point 1 (T1, N = 206) and time point 2 (T2, N = 266). A graphical representation of the results of the unconditional logistic regression used to regress allergic symptoms (yes/no) on Harvey exposures (yes/no). Figure depicts size of odds ratios and level of significance seperately for Low ADI vs. High ADI neighborhoods. [file 12940_2021_694_MOESM4_ESM.docx]

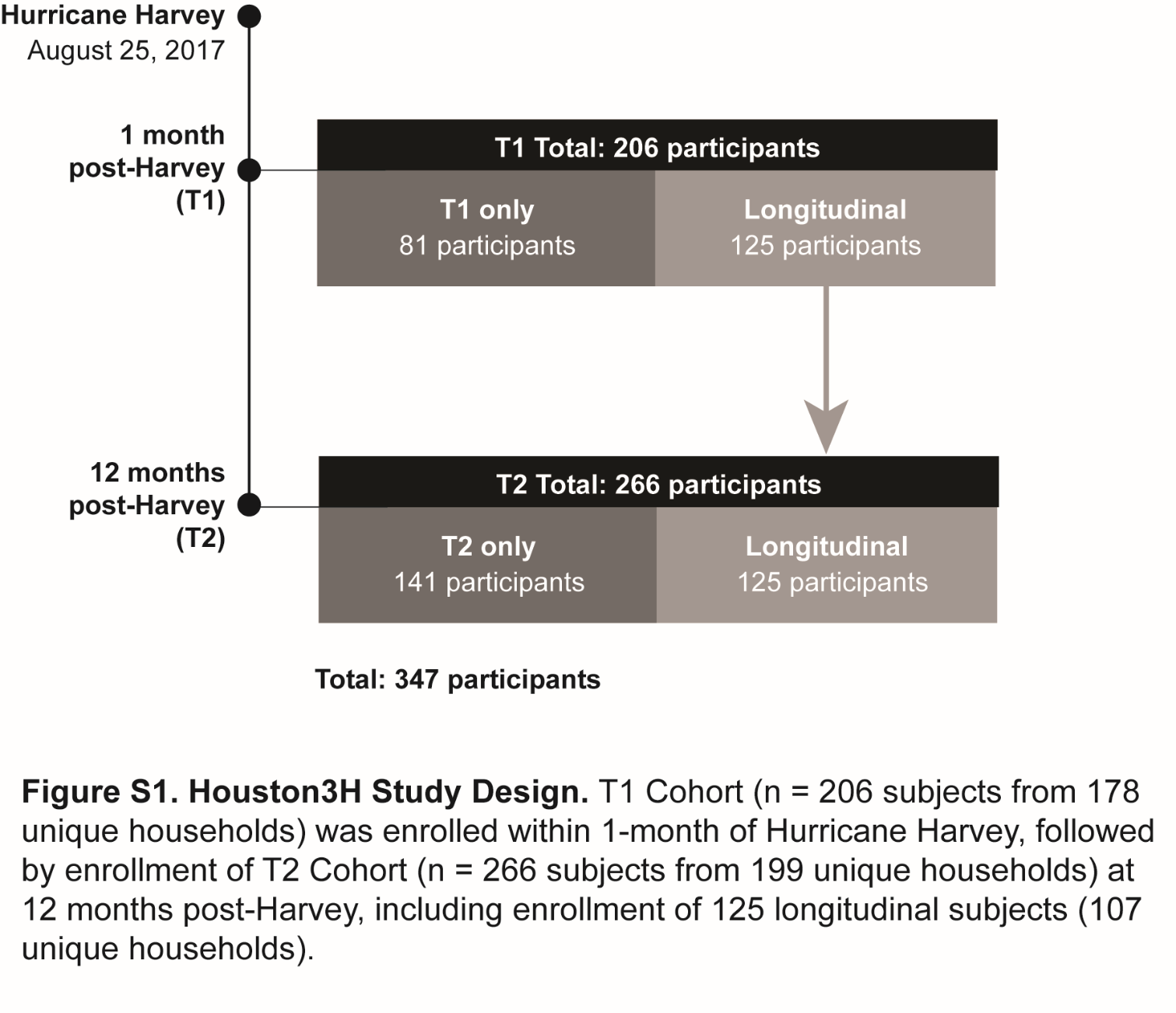


| Figure S1. Houston Hurricane Harvey Health (Houston3H) Study flow of participants’ enrollment and data collection. |
| --- |


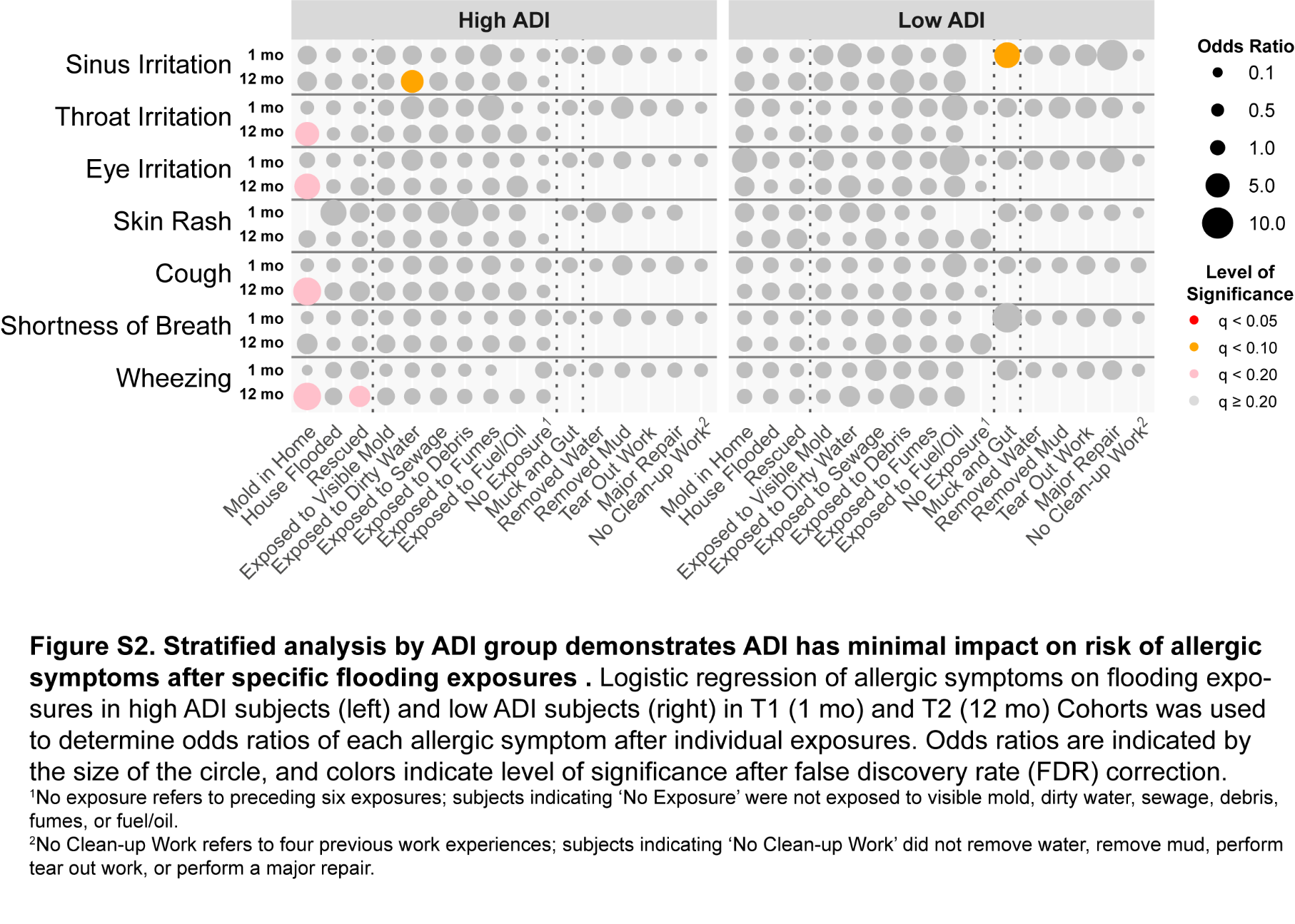


| Figure S2. ADI-stratified logistic regression (Odds Ratio) of allergic symptoms on flooding exposures at time point 1 (T1, N=206) and time point 2 (T2, N=266).  Note (i): Area Deprivation Index (ADI) is a composite measure of “neighborhood” socioeconomic disadvantage. Higher score represents increasing socioeconomic disadvantage.  Note (ii): Odds ratios are indicated by the size of the circle, and colors indicate level of significance after false discovery rate (FDR) correction.  ^1^ No exposure refers to preceding six exposures; subjects indicating ‘No Exposure’ were not exposed to visible mold, dirty water, sewage, debris, fumes, or fuel/oil.  ^2^ No Clean-up Work refers to four previous work experiences; subjects indicating ‘No Clean-up Work’ did not remove water, remove mud, perform tear out work, or perform a major repair. |
| --- |
