# Supplementary material for: Houston hurricane Harvey health (Houston-3H) study: assessment of allergic symptoms and stress after hurricane Harvey flooding
Source: Environ Health. 2021 Jan 19;20:9. doi: 10.1186/s12940-021-00694-2 (PMC7816385; doi:10.1186/s12940-021-00694-2)
Supplement: Supplementary file 5 — Additional file 5. Comparisons of the health outcomes reported by the participants that provided data at both T1 and T2 (N = 125). Several graphs and analysis output tables show the results from the analysis that comapred heath conditions at T1 versus T2 exclusively among the participants with repeated measures (the paired-samples; N = 125). [file 12940_2021_694_MOESM5_ESM.docx]

**SHORTNESS OF BREATH**

| **Hypothesis Test Summary** | | | | |
| --- | --- | --- | --- | --- |
|  | Null Hypothesis | Test | Sig.^a,b^ | Decision |
| 1 | The distributions of different values across T1 Shortness of Breath and T2 Shortness of Breath are equally likely. | Related-Samples McNemar Change Test | .839^c^ | Retain the null hypothesis. |


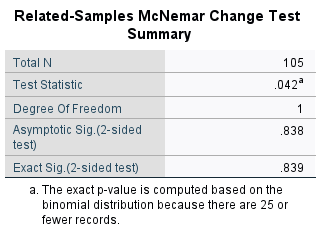


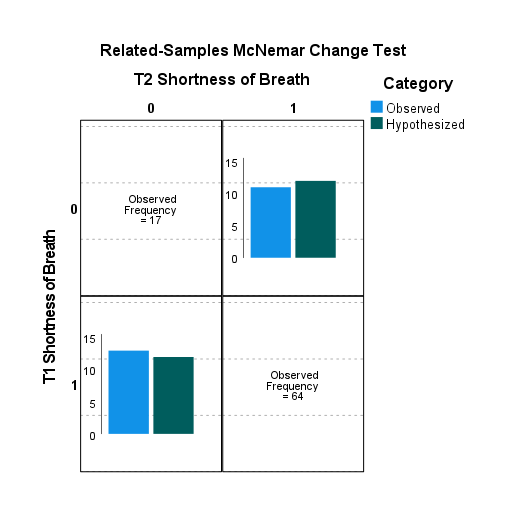


**SHORTNESS OF BREATH**


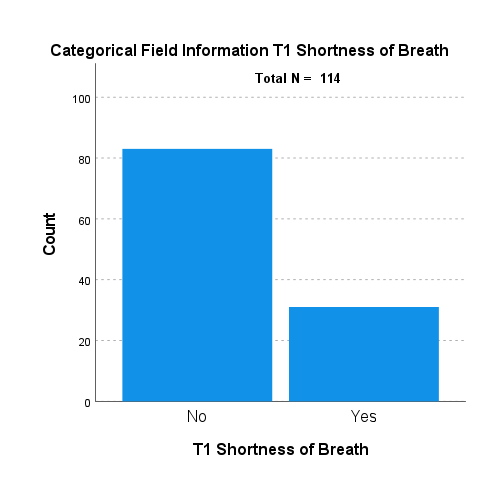


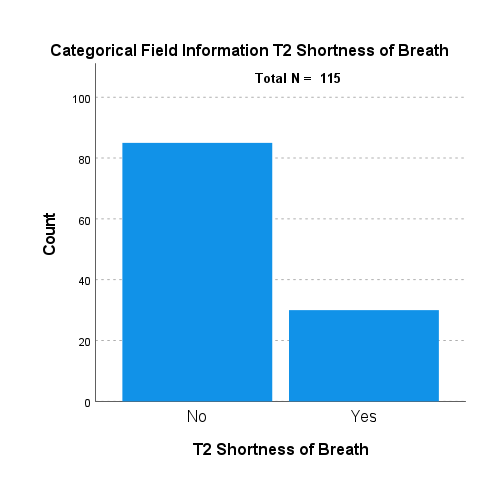


**COUGH**

| **Hypothesis Test Summary** | | | | |
| --- | --- | --- | --- | --- |
|  | Null Hypothesis | Test | Sig.^a,b^ | Decision |
| 1 | The distributions of different values across T1 Cough and T2 Cough are equally likely. | Related-Samples McNemar Change Test | .011^c^ | Reject the null hypothesis. |


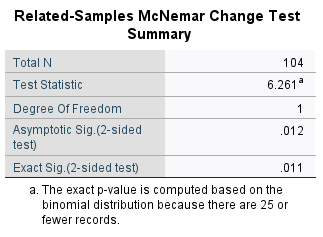


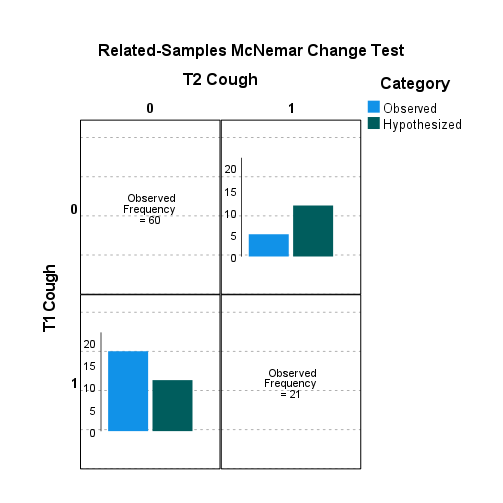


**COUGH**


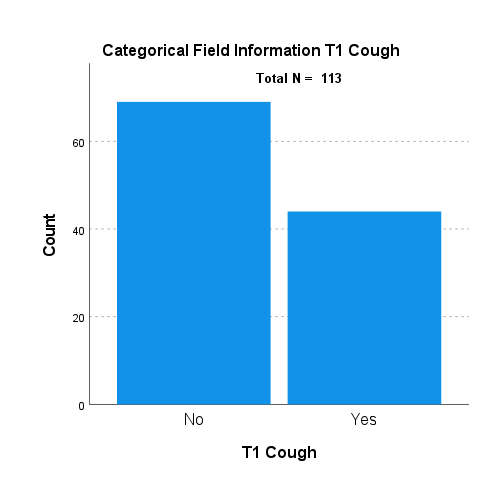


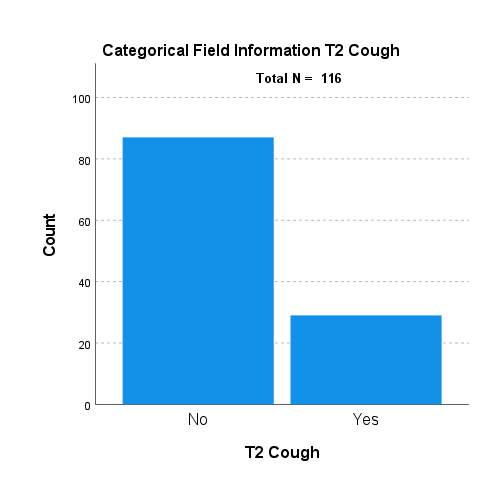


**EYE IRRITATION**

| **Hypothesis Test Summary** | | | | |
| --- | --- | --- | --- | --- |
|  | Null Hypothesis | Test | Sig.^a,b^ | Decision |
| 1 | The distributions of different values across T1 Eye Irritation and T2 Eye Irritation are equally likely. | Related-Samples McNemar Change Test | .009 | Reject the null hypothesis. |


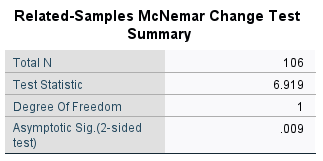


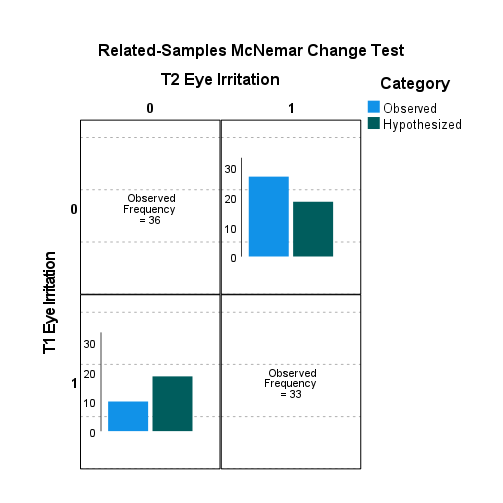


**EYE IRRITATION**


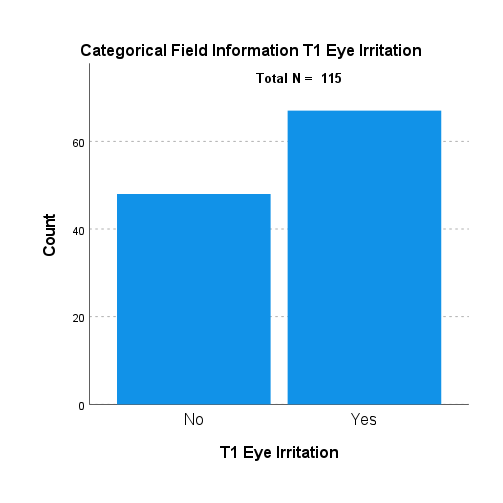


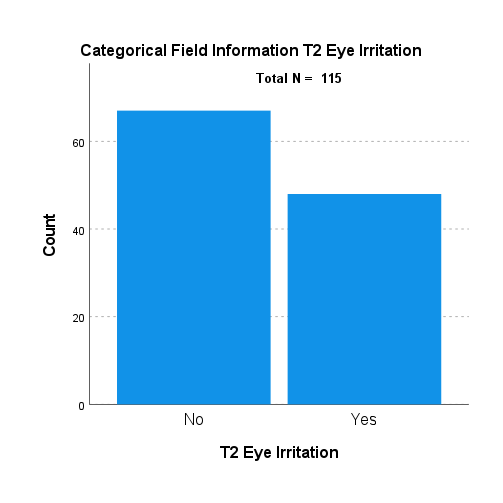


**SINUS IRRITATION**

| **Hypothesis Test Summary** | | | | |
| --- | --- | --- | --- | --- |
|  | Null Hypothesis | Test | Sig.^a,b^ | Decision |
| 1 | The distributions of different values across T1 Sinus Irritation and T2 Sinus Irritation are equally likely. | Related-Samples McNemar Change Test | .026 | Reject the null hypothesis. |


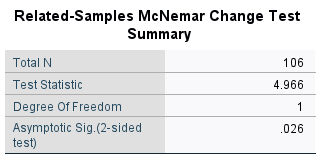


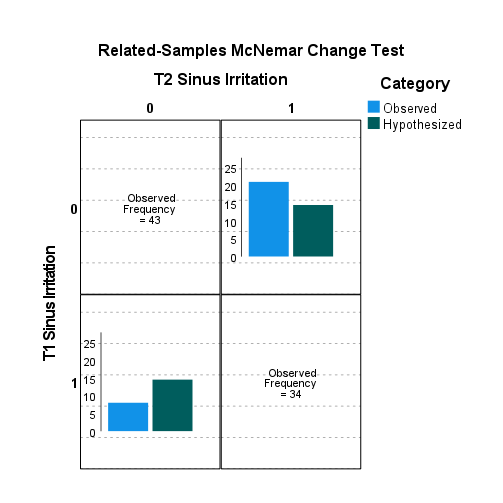


**SINUS IRRITATION**


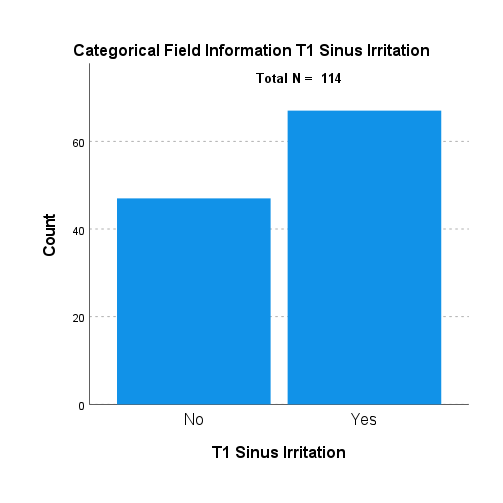


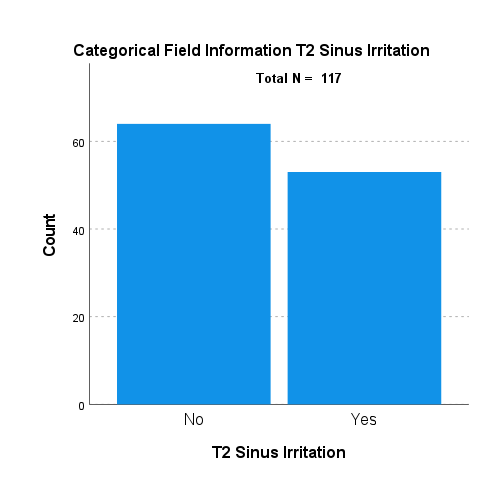


**SKIN RASH**

| **Hypothesis Test Summary** | | | | |
| --- | --- | --- | --- | --- |
|  | Null Hypothesis | Test | Sig.^a,b^ | Decision |
| 1 | The distributions of different values across T1 Skin Rash and T2 Skin Rash are equally likely. | Related-Samples McNemar Change Test | .719 | Retain the null hypothesis. |


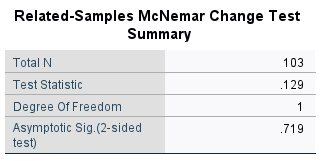


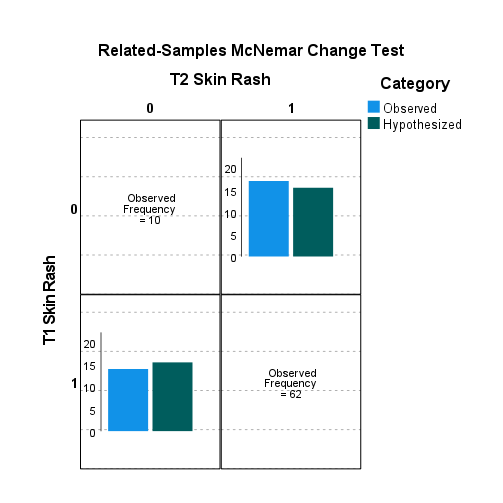


**SKIN RASH**


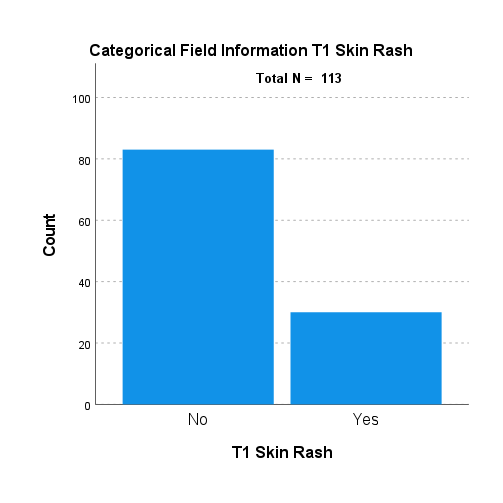


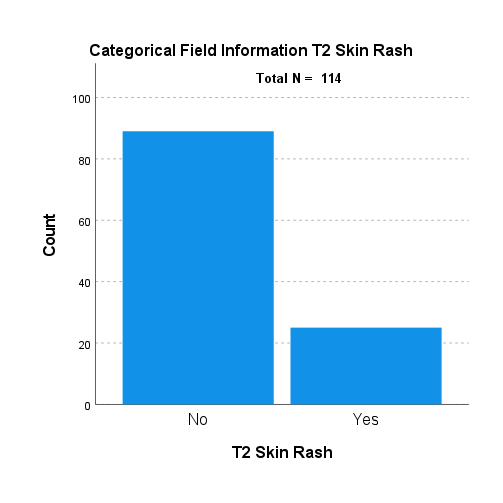


**THROAT IRRITATION**

| **Hypothesis Test Summary** | | | | |
| --- | --- | --- | --- | --- |
|  | Null Hypothesis | Test | Sig.^a,b^ | Decision |
| 1 | The distributions of different values across T1 Throat Irritation and T2 Throat Irritation are equally likely. | Related-Samples McNemar Change Test | .035 | Reject the null hypothesis. |


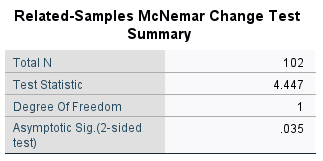


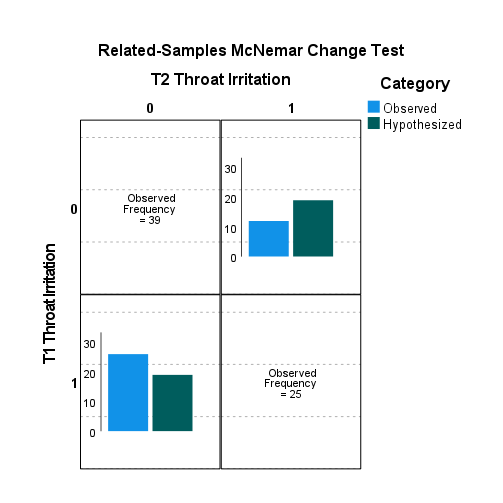


**THROAT IRRITATION**


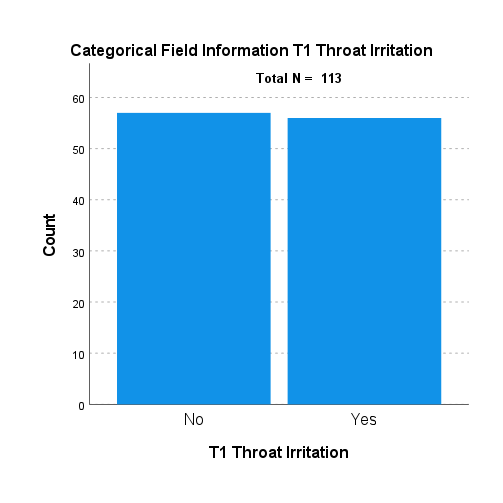


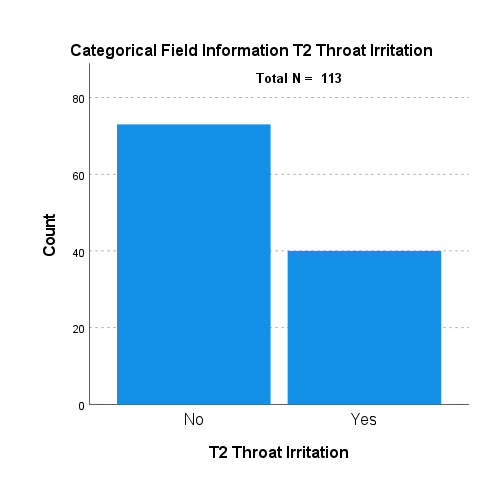


**WHEEZING**

| **Hypothesis Test Summary** | | | | |
| --- | --- | --- | --- | --- |
|  | Null Hypothesis | Test | Sig.^a,b^ | Decision |
| 1 | The distributions of different values across T1 Wheezing and T2 Wheezing are equally likely. | Related-Samples McNemar Change Test | 1.000^c^ | Retain the null hypothesis. |


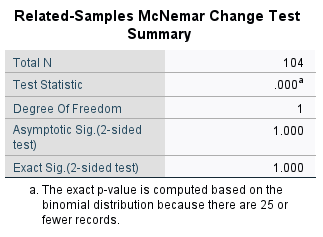


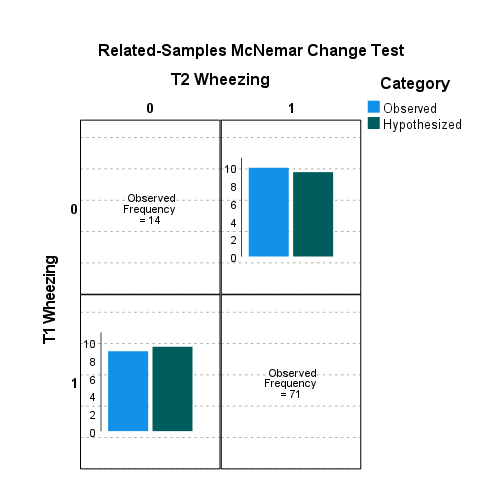


**WHEEZING**


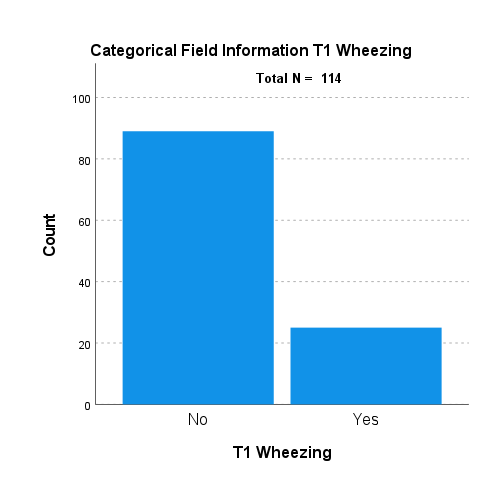


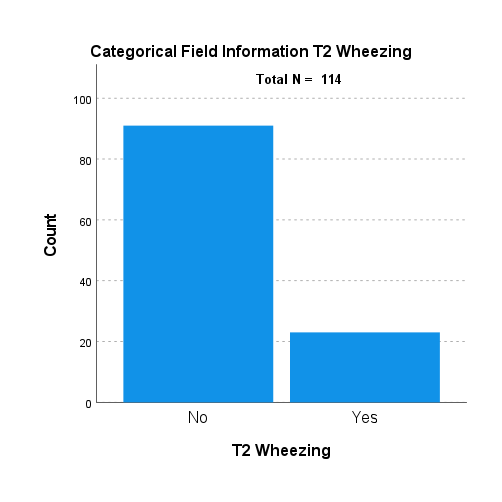


**STRESS LEVEL**

| **Paired Samples Statistics** | | | | | |
| --- | --- | --- | --- | --- | --- |
|  | | Mean | N | Std. Deviation | Std. Error Mean |
| Pair 1 | T2 Stress level | 4.83 | 109 | 3.138 | .301 |
|  | T1 Stress level | 8.15 | 109 | 1.933 | .185 |

| **Paired Samples Test** | | | | | | | | | |
| --- | --- | --- | --- | --- | --- | --- | --- | --- | --- |
|  | | Paired Differences | | | | | t | df | Sig. (2-tailed) |
|  |  | Mean | Std. Deviation | Std. Error Mean | 95% Confidence Interval of the Difference | |  |  |  |
|  |  |  |  |  | Lower | Upper |  |  |  |
| Pair 1 | T2 Stress level - T1 Stress level | -3.321 | 2.997 | .287 | -3.890 | -2.752 | -11.571 | 108 | .000 |

| **Paired Samples Effect Sizes** | | | | | | |
| --- | --- | --- | --- | --- | --- | --- |
|  | | | Standardizer^a^ | Point Estimate | 95% Confidence Interval | |
|  |  |  |  |  | Lower | Upper |
| Pair 1 | T2 Stress level - T1 Stress level | Cohen's d | 2.997 | -1.108 | -1.346 | -.868 |
|  |  | Hedges' correction | 3.007 | -1.104 | -1.341 | -.865 |
| *a. The denominator used in estimating the effect sizes.*  *Cohen's d uses the sample standard deviation of the mean difference.*  *Hedges' correction uses the sample standard deviation of the mean difference, plus a correction factor.* | | | | | | |
